# Exploratory Assessment of the Internal Structure of the Spanish Version of the Persenius Scale for Enteral Nutrition in Intensive Care Units: A Cross-Sectional Psychometric Study

**DOI:** 10.3390/jcm15155987

**Published:** 2026-07-31

**Authors:** Antonio Martínez-Sabater, Vicente Doménech-Briz, Michał Czapla, Raúl Juárez-Vela, Mercedes Sánchez-Barba, Vicente Gea-Caballero, Aurora García-Tejedor, Paula Sánchez-Palomares, Raquel María Martínez-Pascual, Elena Chover-Sierra, José Angel Santos-Sanchez, Jacek Smereka

**Affiliations:** 1 Nursing Care and Education Research Group (GRIECE, GIUV2019-456), Department of Nursing, Faculty of Nursing and Podiatry, University of Valencia, 26004 Valencia, Spainelena.chover@uv.es (E.C.-S.); 2 Care Research Group (INCLIVA), Clinical Hospital of Valencia, University of Valencia, 26004 Valencia, Spain; 3 GRUPAC Research Group in Care, Faculty of Health Sciences, University of La Rioja, 26006 Logroño, Spain; 4 Intensive Care Unit, Ribera University Hospital, Alzira, 46600 Valencia, Spain; 5 Department of Emergency Medical Service, Faculty of Nursing and Midwifery, Wroclaw Medical University, 51-618 Wroclaw, Poland; 6 Institute of Neurosciences of Castile and León (INCYL), Institute of Biomedical Research of Salamanca (IBSAL), University of Salamanca, 37008 Salamanca, Spain; 7 Department of Statistics, Faculty of Medicine, University of Salamanca, 37007 Salamanca, Spain; 8 Faculty of Health Sciences, Valencian International University, 46002 Valencia, Spain; 9 Community Health and Care Research Group (SALCOM), Valencian International University, 46002 Valencia, Spain; 10 Grupo de Bioactividad e Inmunología Nutricional (BIOINUT), Universidad Internacional de Valencia (VIU), 46002 Valencia, Spain; 11 Department of Internal Medicine, General Hospital of Valencia, 460060 Valencia, Spain; 12 Department of Biomedical Sciences, Faculty of Medicine, University of Salamanca, 37007 Salamanca, Spain

**Keywords:** enteral nutrition, intensive care units, critical care nursing, psychometrics, questionnaires, structural validity

## Abstract

**Background/Objectives:** Enteral nutrition is an essential component of care for critically ill patients, and nurses play a key role in its assessment, delivery, and monitoring. Although the Spanish version of the Persenius Scale has previously undergone translation, cross-cultural adaptation, and content validation, its structural validity has not yet been evaluated. Therefore, this study aimed to evaluate the structural validity of the Spanish version of the Persenius Scale in a sample of intensive care unit nurses in Spain. **Methods:** A cross-sectional study was conducted using an online questionnaire administered to intensive care unit nurses. The analytical sample included 329 participants recruited through non-probability snowball sampling. Sampling adequacy was assessed using the Kaiser–Meyer–Olkin index and Bartlett’s test of sphericity. Principal component analysis with Varimax rotation was performed to evaluate the structural validity of the instrument. Hierarchical cluster analysis was also used to examine the coherence of item grouping. **Results:** The Kaiser–Meyer–Olkin value was 0.88, and Bartlett’s test of sphericity was significant, supporting the suitability of the data for component analysis. A five-component solution was retained, explaining approximately 58% of the total variance. The components were organizational resources, specialized training, professional responsibility, perceived competence, and technical modality of enteral administration. Organizational resources and professional responsibility showed the most homogeneous loading patterns, whereas several technical practice items demonstrated higher uniqueness and weaker structural integration. **Conclusions:** The present study provides preliminary evidence regarding the internal structure of the Spanish version of the Persenius Scale by demonstrating a clinically interpretable five-component structure. The instrument may support the assessment of educational, organizational, and professional aspects of enteral nutrition practice among intensive care unit nurses.

## 1. Introduction

Nutritional therapy is a fundamental component of the management of critically ill patients, and its appropriate implementation has been associated with fewer complications, shorter hospital stays, and better clinical outcomes. When the gastrointestinal tract is functional, enteral nutrition (EN) is the preferred route because of its physiological benefits, lower cost, and more favorable outcomes compared with parenteral nutrition [1,2]. However, the delivery of EN in intensive care units (ICUs) remains suboptimal and varies across settings, being influenced by structural, organizational, and professional factors [3]. This issue is especially relevant because malnutrition affects approximately 40–50% of critically ill patients and is associated with increased morbidity, mortality, prolonged hospital stays, and other complications [4].

In this context, ICU nurses play a central role in nutritional assessment, implementation of nutritional therapy, and monitoring of complications [5]. Evidence suggests that knowledge level, perceived professional responsibility, availability of protocols, and specific training directly influence the adequacy of nutritional support [5,6,7]. Nevertheless, previous studies have identified deficiencies in nutritional assessment, variability in clinical practice, and substantial reliance on experience-based learning or informal consultation with colleagues [8], findings that have also been reflected in later studies [6,7,9]. In addition, nutritional care has often been reported to receive lower priority than other ICU interventions [10,11], and the use of standardized protocols remains insufficient in many settings [10,12,13].

Perceived knowledge, understood here as the subjective appraisal of one’s own professional competence, is an important construct for understanding clinical decision-making, adherence to evidence-based practices, and educational needs [14,15]. Previous studies have shown discrepancies between perceived and actual knowledge, as well as the influence of self-perceived competence on the implementation of complex interventions [14,15,16,17]. In the field of clinical nutrition, this dimension is particularly relevant because specific training and professional competence have been associated with better clinical outcomes and higher quality of care [18,19,20,21].

In response to these limitations, several instruments have been developed to assess knowledge, attitudes, and practices related to EN in the ICU, showing acceptable psychometric performance [21,22,23,24,25,26,27,28]. Among them, the questionnaire developed by Persenius et al. is particularly relevant because it was designed to evaluate nurses’ perceptions of enteral nutrition, including aspects such as knowledge sources, professional responsibility, institutional support, and documentation-related resources [8,10,12]. Despite the relevance of nutritional support and the central role of nurses, studies in Spain examining enteral nutrition-related knowledge and perceptions remain scarce. Recently, the Persenius Scale was translated, cross-culturally adapted, and content validated in Spanish, demonstrating adequate semantic equivalence, content validity, and preliminary internal consistency in a pilot sample [23]. However, the previous validation study did not evaluate the structural validity of the questionnaire or whether its underlying construct was preserved in the Spanish ICU nursing population.

Evaluation of structural validity represents a distinct stage of psychometric validation and is essential to determine whether questionnaire items cluster into meaningful latent dimensions corresponding to the intended constructs. Accordingly, the aim of this study was to evaluate the structural validity of the Spanish version of the Persenius Scale by examining its underlying component structure in a sample of ICU nurses in Spain.

## 2. Materials and Methods

### 2.1. Study Design

A cross-sectional observational study was conducted among nurses working in intensive care units (ICUs) in Spain. The study was reported in accordance with the Strengthening the Reporting of Observational Studies in Epidemiology (STROBE) guidelines to enhance transparency and reproducibility.

### 2.2. Participants and Sampling

The study sample consisted of nurses with ICU experience in Spain. Participants were recruited through a non-probability snowball sampling strategy using the Internet and social media platforms, specifically Facebook and Twitter/X, a procedure previously used in similar nursing research [29]. Eligibility criteria included being a registered nurse, having professional experience in an ICU in Spain, and agreeing to participate voluntarily in the study. Sample size was considered adequate for the evaluation of structural validity using exploratory component analysis, based on methodological recommendations suggesting at least 5–7 participants per item [30] A total of 332 nurses responded to the questionnaire. After data screening and exclusion of incomplete questionnaires, the final sample for the structural validity analyses comprised 329 participants. Although this recruitment strategy facilitated access to ICU nurses from different regions of Spain, the use of non-probability snowball sampling may have introduced selection bias and may limit the generalizability of the findings to the broader population of ICU nurses.

### 2.3. Data Collection

Data were collected between July and November 2025 using an online questionnaire administered through the Qualtrics platform. IP control was enabled to reduce the likelihood of duplicate responses. The questionnaire included sociodemographic and professional variables, such as age, sex, academic background, specific training in nutrition, and ICU experience, together with the Spanish-adapted version of the Persenius Scale [8,23].

### 2.4. Instrument

The Persenius Scale was originally developed to assess nurses’ perceptions of enteral nutrition in critical care settings [8]. The Spanish version used in this study had previously undergone translation, cross-cultural adaptation, and content validation [23]. The instrument includes items referring to sources of knowledge about enteral nutrition, perceived professional responsibility, self-perceived competence, availability of organizational and documentary resources, and routine clinical practices related to enteral nutrition administration and monitoring. In the present study, the analysis focused on items 10 to 44, which represent the substantive sections of the questionnaire related to enteral nutrition knowledge sources, responsibility, competence, organizational support, and practice-related aspects. These items were analysed to evaluate the structural validity of the Spanish version of the questionnaire and to identify its underlying component structure in ICU nurses.

### 2.5. Statistical Analysis

Statistical analyses were performed using IBM SPSS Statistics version 26.0 (IBM Corp., Armonk, NY, USA). The psychometric evaluation focused on the assessment of the structural validity of the Spanish version of the Persenius Scale. First, descriptive analyses were conducted using frequencies and percentages for categorical variables and means and standard deviations for continuous or ordinal variables, as appropriate. The proportion of missing data per item was below 5%, and in most items remained below 1–2%. Given the low proportion and dispersed distribution of missing values, listwise deletion was applied for the internal structure analyses, a procedure considered acceptable in studies with limited missing data [31]. The adequacy of the data for component extraction was assessed using the Kaiser–Meyer–Olkin (KMO) measure of sampling adequacy and Bartlett’s test of sphericity, following established recommendations for studies examining the internal structure of health-related instruments [30,31,32,33]. As the original questionnaire had not previously undergone an evaluation of its internal structure, an exploratory approach was considered appropriate. To evaluate the structural validity of the instrument, principal component analysis (PCA) with Varimax rotation was performed. PCA was selected as an exploratory technique to identify clinically interpretable item groupings within the Spanish version of the questionnaire. Component loadings of at least 0.40 were considered to indicate practical relevance. The number of retained components was determined through visual inspection of the scree plot, together with criteria of parsimony and clinical interpretability, given that the Kaiser criterion based solely on eigenvalues greater than 1 may overestimate dimensionality in instruments with a relatively high number of items [30,34,35]. To provide complementary descriptive evidence regarding item grouping, a hierarchical cluster analysis was additionally performed using Ward’s method and Euclidean distance as the measure of proximity. This analysis was used as a supportive approach to examine the coherence of item groupings identified in the rotated component solution [36]. In addition, the distribution of items in the rotated component space was graphically represented to facilitate interpretation of the retained components and to visualize clustering patterns among items. Statistical significance was set at *p* < 0.05.

## 3. Results

### 3.1. Participant Characteristics

The analytical sample comprised 329 ICU nurses in Spain, with a mean age of 36.2 ± 10.7 years (range: 22–65 years). Most participants were women (73.9%). The majority reported at least 2 years of ICU experience (70.9%), and the mean duration of professional experience in the ICU was 9.44 ± 9.01 years. Regarding academic background, 59.9% held a bachelor’s degree or equivalent nursing qualification, 36.2% had a master’s degree, and 4.0% held a doctoral degree. Specific training in nutrition was reported mainly through continuing education activities (85.5%). Participant characteristics are summarized in Table 1.

Regarding organizational resources, 70.0% of participants reported the availability of written protocols for enteral nutrition (EN) management in their unit. However, only 7.0% indicated the presence of a nurse specifically responsible for nutrition, and 17.8% reported the existence of a nutrition team in the ICU. At the hospital level, 78.9% reported the availability of a nutrition team, and 70.6% identified internal professionals who could be consulted about nutrition-related issues, whereas external reference professionals were reported less frequently (18.0%) (Table 2). Decisions regarding EN prescription, including type, amount, and infusion rate, were reported to be made predominantly by physicians (>94). Participation by dietitians-nutritionists was lower (approximately 19–21%), and nurses showed particularly limited involvement, especially in decisions related to infusion rate (13.7%).

### 3.2. Sources of Knowledge and Clinical Practices Related to Enteral Nutrition

Among the reported sources of knowledge on enteral nutrition (EN), consultation with colleagues was the most frequent (3.49 ± 1.05), followed by courses (3.03 ± 1.12) and Internet/Intranet resources (3.01 ± 1.16). Conferences and scientific literature were the least frequently used sources. Mean scores across the main content areas showed a descending pattern, with higher values for responsibility (3.19 ± 0.90), followed by knowledge (2.75 ± 0.86) and documentation (2.40 ± 0.90). The highest responsibility scores were observed for the prevention of complications, whereas the lowest scores corresponded to defining goals and planning nutritional care. With respect to clinical practices, the most frequently reported interventions were flushing the feeding tube after administration of nutrition or medication (4.85 ± 0.46), use of a feeding pump (4.68 ± 0.62), and checking gastric residual volume (4.51 ± 0.91). In contrast, bolus feeding and the organization of feeding schedules to allow nocturnal rest were less frequently reported. Notably, 28% of respondents indicated that medications not intended to be crushed were nevertheless routinely administered in crushed form through the feeding tube. When analyzed according to ICU experience, nurses with less than 2 years of experience showed significantly higher scores in responsibility and documentation (*p* < 0.05), although effect sizes were small to moderate. No relevant differences were observed in perceived knowledge. Results are summarized in Table 3.

### 3.3. Internal Structure of the Instrument

**Figure 1:**
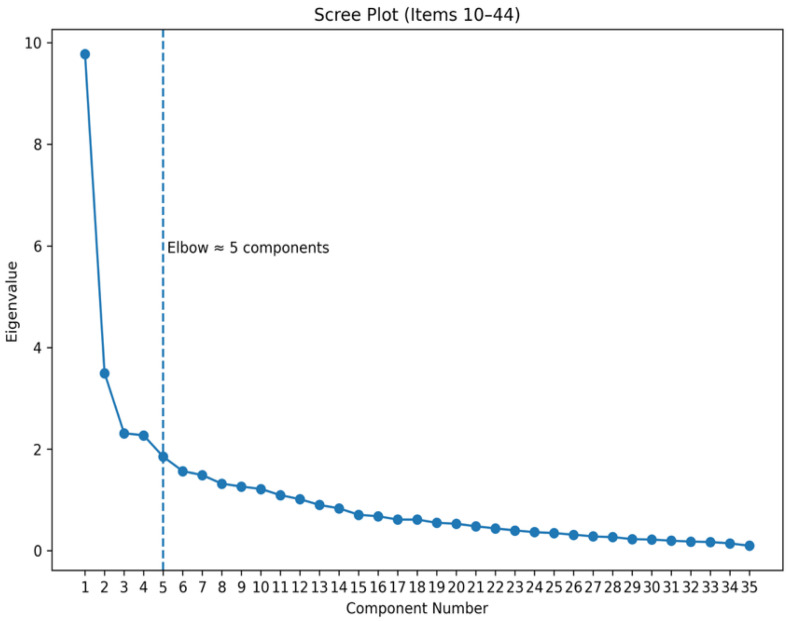
Scree plot for determining the number of retained components.

The internal structure analyses were conducted on a final sample of 329 participants. The proportion of missing data was low across all items, with most variables showing between 326 and 329 valid responses. Given the low proportion and dispersed distribution of missing values, listwise deletion was applied for the component analyses. Sampling adequacy for component extraction was supported by a Kaiser–Meyer–Olkin value of 0.88, indicating very good sampling adequacy. Bartlett’s test of sphericity was statistically significant (chi^2^(595) = 5074.76;\ *p* < 0.001), confirming that the correlation matrix was appropriate for component extraction. The number of retained components was determined by visual inspection of the scree plot. The plot showed a clear inflection point at the fifth component, after which the slope of the eigenvalues progressively stabilized. Although the Kaiser criterion (eigenvalues > 1) suggested retention of a larger number of components, this approach is known to overestimate dimensionality in instruments with a relatively high number of items. Therefore, a five-component solution was retained because it provided the most parsimonious and clinically interpretable representation of the data. The five retained components explained approximately 58% of the total variance. Varimax rotation yielded a component solution considered clinically interpretable. Based on the pattern of item loadings, the retained components were provisionally interpreted as reflecting organizational resources, specialized training, professional responsibility, perceived competence, and technical modality of enteral administration. The components related to organizational resources and professional responsibility showed the strongest and most homogeneous loadings, whereas some items related to technical care displayed greater uniqueness and lower integration within the overall solution. The scree plot is presented in Figure 1.

### 3.4. Rotated Component Solution

Varimax rotation allowed the identification of five conceptually differentiated components. Component loadings of at least |0.40| were used as the main criterion for interpretation. Overall, the rotated solution showed a coherent pattern of item groupings. Detailed component loadings and uniqueness values are presented in Table 4.

#### 3.4.1. Component 1: Organizational Resources

This component grouped items 29–33, which referred to the availability of supporting documentation for the assessment, planning, and implementation of nutritional interventions. Component loadings ranged from 0.797 to 0.857, and uniqueness values were low (0.178–0.276), indicating that a substantial proportion of the variance of these items was explained by the retained solution. This component showed a strong and homogeneous structure.

#### 3.4.2. Component 2: Specialized Training

This component mainly included items 12–15, 17, and 18, related to external and formal learning sources such as conferences, scientific literature, and courses. Component loadings ranged from 0.565 to 0.713. Item 16 (university education) showed its highest loading on this component (0.356), although below the predefined interpretative threshold, suggesting only partial integration within this component. Uniqueness values were moderate for the main items (0.447–0.616), whereas item 16 showed high uniqueness (0.762), indicating weaker representation in the solution. Items 10 and 11 did not show meaningful loadings and presented high uniqueness values (0.733 and 0.765, respectively), reflecting limited structural integration.

#### 3.4.3. Component 3: Professional Responsibility

This component comprised items 19–23, related to responsibility for assessment, goal setting, planning, prevention, and follow-up of nutritional treatment. Component loadings ranged from 0.670 to 0.792, and uniqueness values ranged from 0.300 to 0.540, indicating adequate representation. This component showed a clear and consistent pattern of loadings.

#### 3.4.4. Component 4: Perceived Competence

This component grouped primarily items 24–28, which referred to the perception of having sufficient knowledge to assess nutritional status, define goals, plan and implement nutritional interventions, and prevent complications related to nutritional therapy. Component loadings ranged from 0.535 to 0.648. Uniqueness values for these items were relatively low (0.275–0.314), indicating adequate representation and a homogeneous structure. In addition, item 36 (daily inspection of the nostrils) and item 38 (cleaning of the enteral feeding syringe after use) showed moderate loadings on this component (0.559 and 0.428, respectively), suggesting some association between self-perceived competence and the performance of appropriate clinical practices. However, both items displayed higher uniqueness values (0.607 and 0.784, respectively), indicating weaker structural integration than the core perceived competence items. Item 10 (consultation with colleagues) showed its highest loading on this component (−0.356), although below the interpretative threshold of |0.40|, and showed high uniqueness (0.733), suggesting limited integration within this component.

#### 3.4.5. Component 5: Technical Modality of Enteral Administration

This component included items 39–43, related to modality of administration (continuous or bolus feeding), use of feeding pumps, checking gastric residual volume, and the organization of feeding schedules. Component loadings ranged from 0.588 to 0.741, with opposite signs in some items due to component orientation. Uniqueness values ranged from 0.439 to 0.600, indicating acceptable representation, although with greater variability than in the more clearly defined components.

Items 34, 35, 37, and 44 did not show meaningful loadings on any retained component, with the highest observed loading for item 35 remaining below the interpretative threshold (0.372). These items also presented high uniqueness values (0.832–0.937), suggesting limited representation within the retained solution.

#### 3.4.6. Graphical Representation and Cluster Analysis

**Figure 2:**
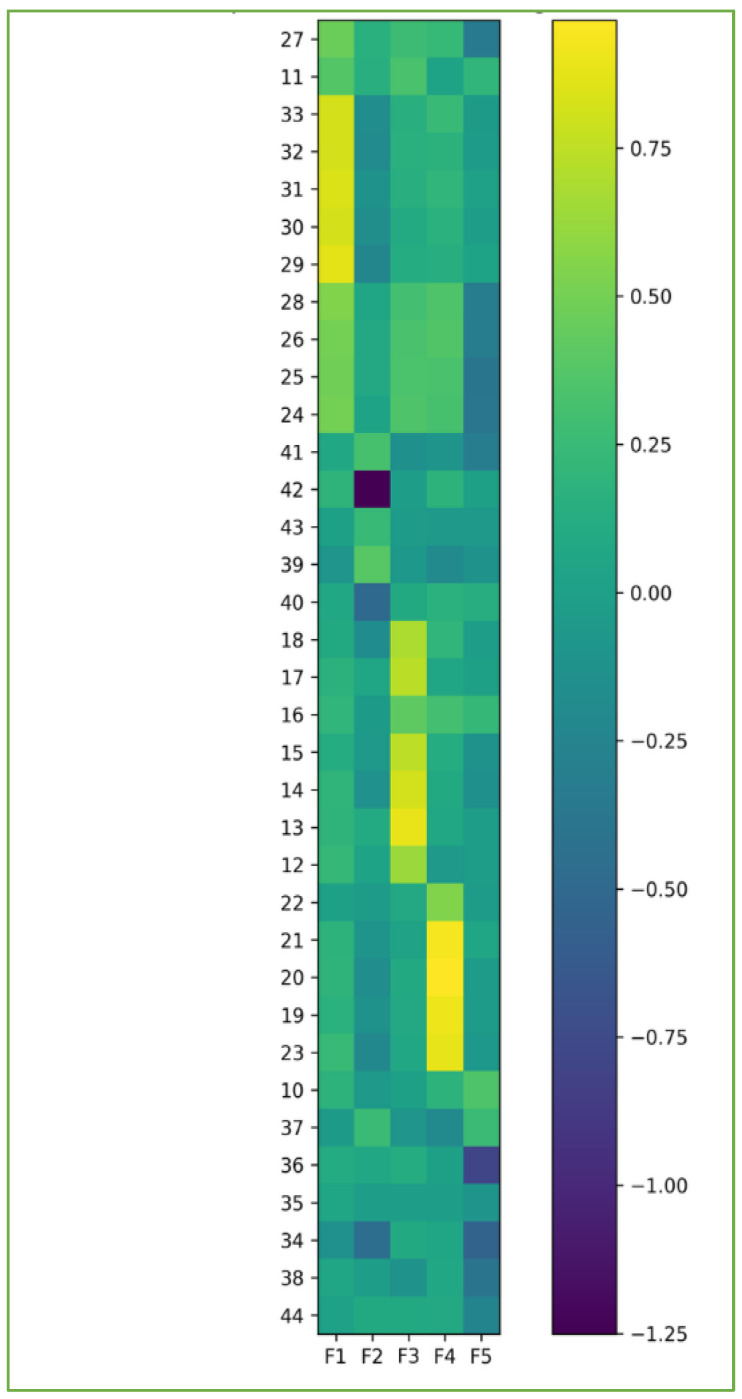
Heatmap of the rotated loadings.

The heatmap of the rotated loadings showed a clear visual grouping of items into five blocks, broadly corresponding to organizational resources, professional responsibility, specialized training, perceived competence, and technical aspects of enteral nutrition administration. The components related to organizational resources and professional responsibility displayed the strongest and most homogeneous loading patterns, whereas some items related to specific clinical practices showed greater dispersion and lower intensity, in line with their higher uniqueness values. This graphical representation was consistent with the component structure identified in the principal component analysis Figure 2.

**Figure 3:**
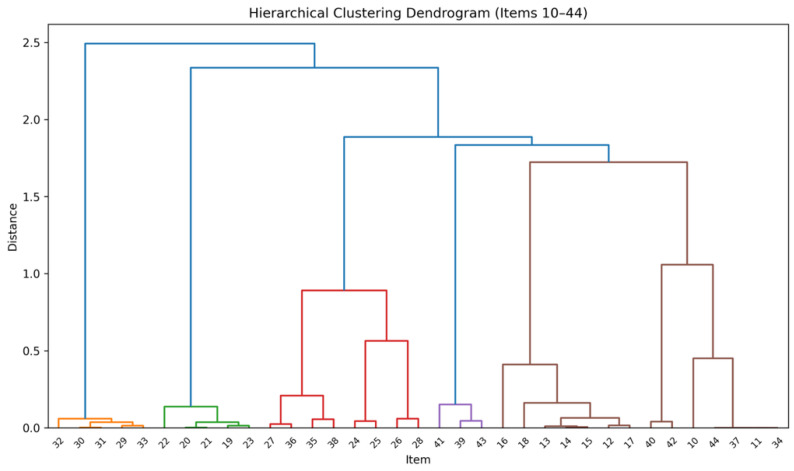
Hierarchical cluster analysis using Ward’s method.

A hierarchical cluster analysis using Ward’s method was conducted on the rotated component loadings. The resulting dendrogram showed item groupings that were generally compatible with the structure identified in the principal component analysis. Items corresponding to organizational resources, professional responsibility, and perceived competence tended to cluster coherently, supporting the interpretability of the retained component solution (Figure 3).

However, the dendrogram did not reproduce the five-component solution exactly. This discrepancy is not unexpected, as cluster analysis is based on overall distances between items and does not distinguish between common and specific variance. In particular, items with high uniqueness values, such as items 34, 35, 37, 38, and 44, showed less clearly defined grouping patterns or were incorporated later in the dendrogram, consistent with their weaker representation in the retained component solution. To complement interpretation of the component solution, items 10–44 were represented in the rotated component space according to their Varimax-rotated loadings. These plots allowed visualization of the grouping of items around the retained components and facilitated identification of structural patterns consistent with the component solution. Specifically, items 29–33 clustered around the axis corresponding to organizational resources, items 19–23 around professional responsibility, items 12–15, 17, and 18 around specialized training, items 24–28 around perceived competence, and items 39–43 around technical aspects of enteral nutrition administration. Item 16 showed a weaker proximity to the specialized training cluster, in line with its lower loading and higher uniqueness. These graphical representations are presented in Supplementary Materials—Figures S1–S3.

## 4. Discussion

The aim of this study was to evaluate the structural validity of the Spanish version of the Persenius Scale in a sample of ICU nurses in Spain. The findings suggest a preliminary five-component solution composed of organizational resources, specialized training, professional responsibility, perceived competence, and technical aspects of enteral nutrition administration, which requires further psychometric verification. Overall, the findings provide preliminary evidence regarding the internal structure of the Spanish version of the instrument by identifying a coherent and clinically interpretable multidimensional solution. One of the main findings of this study was the identification of a coherent and clinically interpretable component structure, providing preliminary evidence regarding the internal structure of the Spanish version of the Persenius Scale. In particular, the components related to organizational resources and professional responsibility showed the most robust and homogeneous configuration, with high loadings and relatively low uniqueness values. This suggests that these components are clearly represented within the Spanish version of the scale and may constitute central dimensions in the assessment of enteral nutrition practices among ICU nurses. Collectively, these results are consistent with previous literature showing that the availability of protocols, documentation, and institutional support, together with clearly assumed professional responsibilities, are key determinants of the quality and consistency of nutritional care in critical care environments [10,12,21]. The component related to specialized training also showed an interpretable structure, although with greater heterogeneity than the organizational resources and professional responsibility components. Items referring to formal and external sources of learning, such as courses, scientific literature, and conferences, tended to load on the same component, suggesting that this component reflects a more structured and academically oriented form of knowledge acquisition. However, some sources of knowledge, particularly university education and consultation with colleagues, were less clearly integrated into the retained solution. This may indicate that learning about enteral nutrition in ICU nursing is shaped by both formal and informal processes, which do not necessarily form a single, clearly bounded construct. An alternative explanation is that some of these items may reflect a different underlying construct rather than the same latent dimension measured by the remaining items of the scale. Further psychometric evaluation is therefore needed to clarify their conceptual relevance and structural role within the instrument. This interpretation is consistent with previous reports indicating that ICU nurses often rely heavily on experiential learning and peer consultation in areas where structured nutrition education may be limited [8,9]. These findings may have practical implications for the design of educational interventions, suggesting that formal training programmes should complement, rather than replace, experiential learning in ICU settings. Professional responsibility and perceived competence emerged as distinct but related components. This distinction is conceptually relevant, as feeling responsible for nutritional care is not necessarily equivalent to perceiving oneself as sufficiently competent to perform it. The separation of these components in the present study supports the idea that responsibility and competence represent different aspects of professional role perception. Previous studies have emphasized that self-perceived competence influences clinical decision-making and implementation of evidence-based practices, but may not always correspond to objective knowledge or actual performance [14,15,16,17]. In this regard, the present findings suggest the value of assessing these domains separately when examining nursing-related component that may affect the quality of nutritional care. The weaker internal coherence of this component should not necessarily be interpreted as a limitation of the instrument. Rather, it may reflect the inherent complexity of enteral nutrition practices in the ICU, where technical procedures are influenced by organizational policies, local routines, patient characteristics, available resources, and multidisciplinary decision-making. This was reflected in the greater variability of component loadings and the higher uniqueness values observed for several technical practice items. Such a pattern may be explained by the fact that technical practices are influenced not only by perceptions or knowledge, but also by local protocols, unit routines, patient characteristics, resource availability, and interprofessional decision-making. Therefore, these items may represent a more context-dependent dimension of nutritional care. Consequently, some degree of heterogeneity within this component may be expected when the instrument is applied across different ICU settings.

The weaker integration of some practice-related items is also consistent with the observation that certain procedures, such as bolus feeding or the scheduling of feeding to allow nighttime rest, may vary substantially across units. The cluster analysis should be interpreted primarily as an illustrative and descriptive tool that facilitated visualization of the item groupings identified by the principal component analysis, rather than as an independent source of psychometric evidence supporting the proposed component structure. Although the dendrogram did not reproduce the five-component solution exactly, it showed coherent clustering patterns for items related to organizational resources, professional responsibility, and perceived competence. This partial convergence is methodologically plausible, since cluster analysis relies on overall distances between items and does not distinguish between common and specific variance in the same way as component-based techniques. Therefore, rather than representing a contradiction, the clustering results may be interpreted as supporting the presence of relatively stable groupings in the instrument while also highlighting the more singular behavior of certain technical items.

From a substantive perspective, the descriptive findings also provide relevant information about enteral nutrition practices in Spanish ICUs. Most participants reported the presence of written enteral nutrition protocols in their units, but the availability of a nurse specifically responsible for nutrition and the existence of ICU nutrition teams were much less common. These descriptive findings are consistent with the domains identified during the structural analysis and provide additional clinical context for interpreting the results. In addition, physicians remained the main professionals responsible for determining the type, amount, and rate of enteral nutrition, with only limited participation from nurses and dietitians-nutritionists. Collectively, these results suggest that nutritional care in Spanish ICUs continues to be organized primarily under a medically led model, with variable levels of interprofessional collaboration. This is important because previous studies have shown that the implementation of protocols, the presence of support structures, and clearer professional roles may contribute to improving the adequacy of nutritional care [10,13,37]. The finding that consultation with colleagues was the most frequent source of knowledge also deserves attention. Although peer exchange is a common and often valuable mechanism of learning in clinical settings, heavy reliance on informal sources may indicate insufficient access to structured or evidence-based educational resources. The relatively lower use of scientific literature and conferences may reflect barriers such as time constraints, limited training opportunities, or the lower prioritization of nutritional care within ICU practice. Together, these findings highlight the need to strengthen formal educational strategies, improve organizational support, and promote multidisciplinary approaches to enteral nutrition in critical care nursing.

### Strengths and Limitations

This study has several limitations that should be considered when interpreting the findings. First, its cross-sectional design precludes conclusions regarding temporal stability or causal relationships. Second, participants were recruited using a non-probability snowball sampling strategy, which may limit the representativeness of the sample and introduce selection bias. Third, data were collected through self-report questionnaires, making the results susceptible to response and social desirability biases. From a psychometric perspective, the present study focused on the evaluation of structural validity using principal component analysis. Although this represents an important stage in the validation process, the absence of confirmatory factor analysis, common factor analysis, assessment of component stability, and evaluation of additional psychometric properties constitutes an important methodological limitation of the present study. These properties should therefore be examined in future studies to provide a more comprehensive psychometric evaluation of the Spanish version of the Persenius Scale. Additionally, the use of non-probability snowball sampling through professional social media platforms may have introduced selection bias. Nurses who were more professionally active, academically engaged, or interested in enteral nutrition may have been more likely to participate, potentially limiting the representativeness of the sample. Consequently, the findings should be interpreted with caution and may not be fully generalizable to the entire population of ICU nurses in Spain. Although the present study provides preliminary exploratory evidence regarding the internal structure of the Spanish version of the Persenius Scale, it represents only one stage of the validation process. Additional psychometric properties, including test–retest reliability, measurement error, construct validity, criterion validity, responsiveness, and cross-cultural measurement invariance, should be evaluated in future studies to provide a more comprehensive assessment of the instrument in accordance with COSMIN recommendations. Although the five-component solution was supported by visual inspection of the scree plot together with considerations of parsimony and clinical interpretability, parallel analysis was not performed. Therefore, the proposed five-component solution should be regarded as preliminary and interpreted with caution until confirmed using more robust dimensionality assessment methods. Future studies should therefore confirm the number of retained components using more robust component-retention procedures, such as parallel analysis. Future validation studies should also evaluate the proposed component structure using common factor analysis with appropriate oblique rotation methods (e.g., Promax or Oblimin), followed by confirmatory factor analysis in an independent sample. Because the identified components are considered exploratory rather than definitive subscales, component-level internal consistency indices were not evaluated in the present study. Before recommending the use of separate subscale scores, future studies should assess their reliability using Cronbach’s alpha, McDonald’s omega, and corrected item–total correlations. Accordingly, the present findings should be interpreted as preliminary evidence regarding the internal structure of the Spanish version of the Persenius Scale rather than as definitive confirmation of its dimensionality.

Despite these limitations, the study has important strengths, including a relatively large national sample of ICU nurses and the systematic evaluation of the structural validity of a previously translated and cross-culturally adapted instrument, providing an important step toward its broader application in Spanish critical care settings. To our knowledge, this is the first study to evaluate the structural validity of the Spanish version of the Persenius Scale in a large national sample of ICU nurses.

## 5. Conclusions

The present study provides preliminary evidence regarding the internal structure of the Spanish version of the Persenius Scale by identifying a coherent and clinically interpretable five-component solution in a sample of ICU nurses in Spain. The exploratory analysis identified five clinically relevant components related to enteral nutrition practice and perception: organizational resources, specialized training, professional responsibility, perceived competence, and technical aspects of enteral nutrition administration. These findings provide preliminary evidence regarding the internal structure of the instrument and suggest that it may be useful for assessing educational, organizational, and professional dimensions associated with enteral nutrition in intensive care settings. Future studies should confirm the observed structure using confirmatory factor analysis in independent samples before broader application and should also examine additional psychometric properties, including internal consistency by component, test–retest reliability, and sensitivity to change. Overall, the Spanish version of the Persenius Scale appears to be a promising instrument for research and for assessing educational, organizational, and professional aspects of enteral nutrition practice in Spanish intensive care units; however, the proposed five-component structure requires further psychometric verification before separate subscale scores can be recommended for routine use.

## Figures and Tables

**Table 1:** Sociodemographic and professional characteristics of the study participants.

Characteristics of the Participants	Mean. Standard Deviation	*n*	(%)
Age	36.2 ± 10.7	-	-
Sex	-	-	-
Women	-	243	73.9%
Men	-	86	26.1%
Professional experience in the ICU	9.44 ± 9.01	-	-
More than two years of experience.		234	70.9%
Academic Background	-	-	-
Graduate	-	197	59.9%
Master	-	119	36.2%
Doctorate	-	13	4%

**Table 2:** Organizational Resources for Enteral Nutrition in Intensive Care Units.

Item	Yes *n* (%)	No *n* (%)	Do Not Know *n* (%)	Valid Responses	Missing Values
1. Are there written guidelines/protocols in your unit on the management of enteral nutrition?	229 (70.0%)	55 (16.8%)	43 (13.1%)	327	2
2. Is there a nurse responsible for nutrition in your unit?	23 (7.0%)	279 (85.3%)	25 (7.6%)	327	2
3. Is there a nutrition team in your unit?	58 (17.8%)	235 (72.1%)	33 (10.1%)	326	3
4. Is there a nutrition team in the hospital?	258 (78.9%)	21 (6.4%)	48 (14.7%)	327	2
5. Are there designated staff members in the hospital whom you can consult about nutrition-related issues?	231 (70.6%)	32 (9.8%)	64 (19.6%)	327	2
6. Are there external professionals outside the hospital whom you can consult about nutrition-related issues?	59 (18.0%)	77 (23.5%)	192 (58.5%)	328	1

**Table 3:** Descriptive results for knowledge sources and enteral nutrition practices.

Variable	Mean * ± SD
Nurses’ sources of knowledge about enteral nutrition	
10. Consulting colleagues	3.49 ± 1.05
17. Courses	3.03 ± 1.12
18. Intranet and Internet	3.01 ± 1.16
16. University education	2.73 ± 1.15
11. On-the-job training	2.67 ± 1.11
13. Specialized training	2.47 ± 1.23
14. Scientific journal articles	2.35 ± 1.13
15. Other literature	2.26 ± 1.07
12. Conferences	1.83 ± 1.02
Nurses’ interventions related to enteral nutrition	
34. Do you flush the feeding tube before administering nutrition or medications?	4.21 ± 1.18
35. Do you flush the feeding tube after administering nutrition or medications?	4.85 ± 0.46
36. Do you inspect the nostrils daily?	4.05 ± 1.11
37. Are medications that should not be crushed administered in crushed form through the feeding tube?	2.58 ± 1.20
38. Do you clean the enteral feeding syringe after each use?	4.37 ± 0.99
39. Is continuous feeding used?	4.16 ± 0.81
40. Is bolus feeding used?	2.10 ± 0.93
41. Do you check gastric residual volume?	4.51 ± 0.91
42. Does the feeding schedule allow for overnight rest?	2.81 ± 1.42
43. Do you use a feeding pump?	4.68 ± 0.62
44. Do you confirm correct nasogastric tube placement before enteral administration?	4.43 ± 0.98

* Footnote: Scores ranged from 1 (“to a very small extent”) to 5 (“to a very great extent”).

**Table 4:** Rotated component loadings and uniqueness values for the Spanish version of the Persenius Scale.

Item	Comp 1	Comp 2	Comp 3	Comp 4	Comp 5	Uniqueness
10				−0.356		0.733
11						0.765
12		0.673				0.517
13		0.713				0.473
14		0.706				0.447
15		0.702				0.456
16		0.356				0.762
17		0.658				0.527
18		0.565				0.616
19			0.773			0.335
20			0.790			0.300
21			0.792			0.324
22			0.670			0.540
23			0.759			0.329
24				0.605		0.314
25				0.648		0.275
26				0.578		0.294
27				0.535		0.492
28				0.567		0.290
29	0.838					0.241
30	0.857					0.193
31	0.856					0.178
32	0.797					0.276
33	0.825					0.184
34						0.933
35				0.372		0.832
36				0.559		0.607
37						0.937
38				0.428		0.784
39					0.696	0.454
40					−0.642	0.542
41					0.588	0.600
42					−0.602	0.557
43					0.741	0.439
44						0.850

## Data Availability

The data supporting the findings of this study are available from the first author upon reasonable request.

## Supplementary Materials

The following supporting information can be downloaded at: https://www.mdpi.com/article/10.3390/jcm15155987/s1, Figure S1. Items in the rotated component space (Components 1 and 2). Figure S2. Items in the rotated component space (Components 3 and 4). Figure S3. Items in the rotated component space (Components 1 and 5).
