# Supplementary material for: Exploratory Assessment of the Internal Structure of the Spanish Version of the Persenius Scale for Enteral Nutrition in Intensive Care Units: A Cross-Sectional Psychometric Study
Source: J Clin Med. 2026 Jul 31;15(15):5987. doi: 10.3390/jcm15155987 (PMC13466332; doi:10.3390/jcm15155987)
Supplement: Supplementary file 1 [file jcm-15-05987-s001.zip › jcm-4442996-supplementary.pdf]

Suplementary

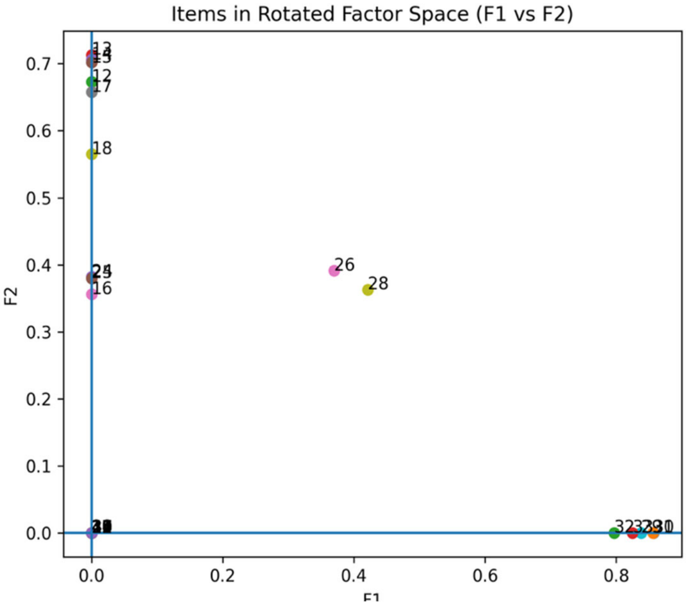

Figure S1 - F3 vs F4 Graph 4. Items in the rotated component space (Components 1 and 2)

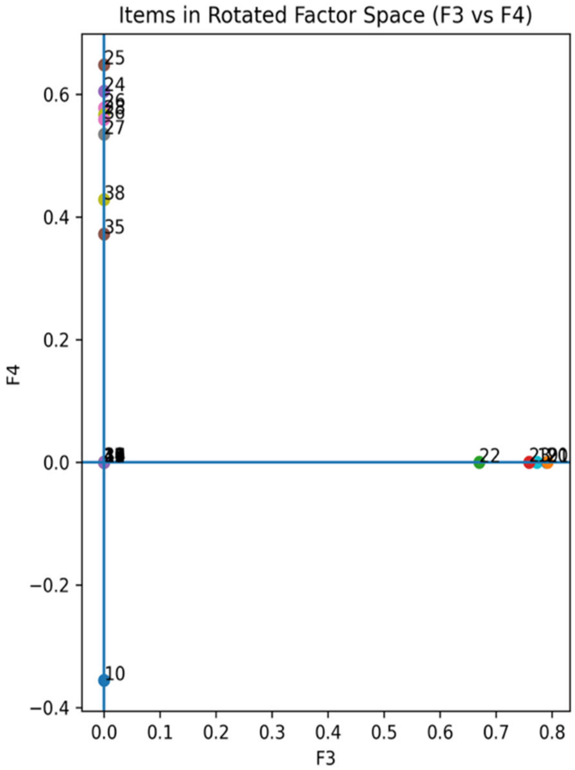

Figure S2. Items in the rotated component space (Components 3 and 4)

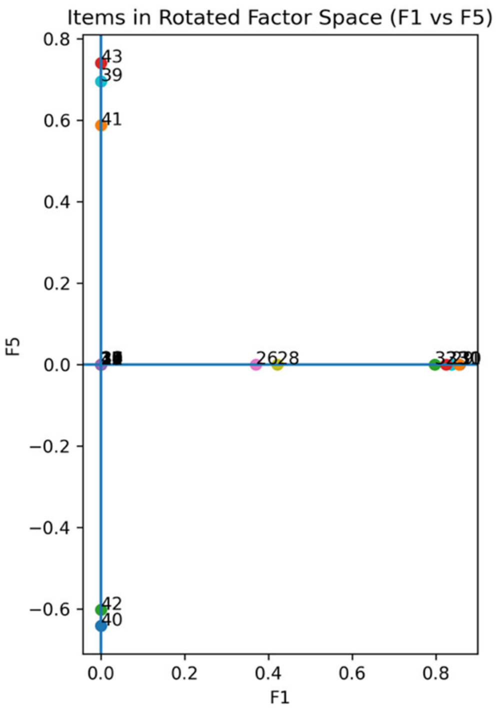

Figure S3. Items in the rotated component space (Components 1 and 5)
